# Trans-esophageal Echocardiographic Assessment of Left Atrial and Left Atrial Appendage Function in Atrial Fibrillation and Rheumatic Heart Disease

**DOI:** 10.7759/cureus.18653

**Published:** 2021-10-11

**Authors:** Rajat Jain, Puneet Aggarwal, Mukesh J Jha, Bhagya Narayan Pandit, Preeti Gupta, Hermohander S Isser

**Affiliations:** 1 Cardiology, Healing Touch Superspeciality Hospital, Ambala, IND; 2 Cardiology, Atal Bihari Vajpayee Institute of Medical Sciences (ABVIMS) and Dr. Ram Manohar Lohia (RML) Hospital, Delhi, IND; 3 Cardiology, Sri Aurobindo Institute of Medical Sciences, Indore, IND; 4 Cardiology, Vardhman Mahavir Medical College and Safdarjung Hospital, Delhi, IND

**Keywords:** left atrial appendage, left atrial function, trans-esophageal echocardiogram, atrial fibrillation, rheumatic heart disease

## Abstract

Introduction: Rheumatic heart disease (RHD) is one of the most typical causes of atrial fibrillation in developing countries like India. The left atrial and left atrial appendage structure and function are deranged in atrial fibrillation and are a major source of thromboembolism. The goal of this study was to assess the left atrial and left atrial appendage function by transesophageal echocardiography in patients with atrial fibrillation and their comparison in patients with or without RHD.

Methods: A total of 172 consecutive patients with atrial fibrillation with or without RHD were subjected to trans-esophageal echocardiography to assess and compare left atrial (LA) and left atrial appendage (LAA) function.

Results: Out of 172 patients with atrial fibrillation, 100 were female (58.1%) and 72 were male (48.9%). The mean age was 54.11±12.3 years, and rheumatic heart disease (RHD) was the commonest cause of atrial fibrillation found in 121 (70.3%) patients. The mean left atrium diameter was significantly higher in RHD patients than in Non-RHD patients (52.08±10.13 vs. 46.67±6.78 mm, p=0.001). Mean left atrial ejection fraction was significantly lower in RHD patients as compared to Non-RHD patients (33.53±5.06 vs. 35.49±5.40%, p=0.024). The mean LAA orifice area of RHD patients was significantly higher than the Non-RHD patients (7.52±1.22 vs 6.94±1.17 mm^2^, p=0.005). Mean LAA emptying velocity was significantly lower in RHD patients than Non-RHD (20.49±3.95 vs. 22.8±5.96 ml/s, p=0.002).

Conclusion: Rheumatic heart disease is still a common cause of atrial fibrillation in developing countries. LA and LAA function is impaired in atrial fibrillation, more in patients with rheumatic heart disease.

## Introduction

With the widespread population aging, the incidence and prevalence of atrial fibrillation (AF) are on the verge of reaching epidemic proportions. The worldwide age-adjusted prevalence of atrial fibrillation, as estimated in the 2010 Global Burden of Disease Study, is 596 per 100,000 men and 373 per 100,000 women [1]. However, in developing countries like India, rheumatic heart disease is still one of the common causes of atrial fibrillation. The left atrium (LA) plays an important role in cardiovascular performance, contributing up to 30% in left ventricular stroke volume [2]. Left atrial and left atrial appendage structure and function are deranged in atrial fibrillation and are a major source of thromboembolism causing significant mortality and morbidity. Only a few studies have addressed left atrial and left atrial appendage (LAA) function in atrial fibrillation and none of the studies (as per our knowledge) has compared the left atrial and left atrial appendage function in atrial fibrillation with or without rheumatic heart disease. To address these lacunae we planned the present study to ascertain impairment of left atrial and left atrial appendage function in atrial function and their comparison in atrial fibrillation with or without rheumatic heart disease.

## Materials and methods

Study design

This was a prospective, observational, analytical study conducted over a period from December 2015 to April 2017 in the Department of Cardiology at a tertiary care center in India. The study included all adult patients with atrial fibrillation diagnosed by irregular pulse on examination and absence of P wave on the electrocardiogram. Exclusion criteria were: any history of a cerebrovascular event or transient ischaemic attack, presence of thrombus in the left atrium, or left atrial appendage on transesophageal echocardiography. A total of 172 patients with atrial fibrillation were included in this study. A well-informed consent was obtained from each patient and the study protocol conforms to the ethical guidelines of the 1975 Declaration of Helsinki as reflected in the prior approval granted by the institution's human research committee. Prior approval was taken by the local institutional ethical committee of Vardhman Mahavir Medical College, Delhi, with approval no 119/2016. The abstract of the study has already been presented at the national meeting of the Cardiology Society of India (2017) [3].

Echocardiogram

A transesophageal echocardiogram was done to assess the valvular lesions and left atrial/left atrial appendage function. Left atrial function was assessed by left atrial diameter, minimum volume, maximum volume, and emptying or ejection fraction. Left atrial appendage function was assessed by LAA orifice area and LAA Doppler velocities. The left atrium and left atrial appendage were assessed by transesophageal echocardiography (TEE) echo in mid esophageal position at 0, 45, 90, and 135-degree views. The patients were divided into those with rheumatic heart disease (RHD) and without rheumatic heart disease (Non-RHD), as per the World Heart Federation echocardiographic criteria for diagnosis of valvular heart disease [4]. Left atrial and left atrial appendage functions were compared in patients with and without RHD.

Statistical analysis

Patients with atrial fibrillation and rheumatic heart disease were compared with patients having atrial fibrillation without valvular heart disease. Normally distributed continuous variables were expressed as mean±SD and the categorical data were presented as proportions. For the comparison of continuous variables between the two groups namely RHD and non-RHD atrial fibrillation, the student’s t-test was used. Similarly, the comparison for skewed data was done by the Mann-Whitney test. To see the association between two categorical variables, Chi-square/Fisher’s exact test was used. A p-value of <0.05 was taken as significant.

## Results

Out of 172 patients, 72 (41.9%) were males and 100 (58.1%) were females. The mean age of the patients was 54.11 ± 12.3 years. The distribution of patients among different age groups was as shown in Table 1. Based on echocardiography, 121 (70.3%) patients had rheumatic heart disease while 51 patients had no evidence of rheumatic heart disease.

**Table 1:** Age distribution of the study population RHD- Rheumatic heart disease, Non-RHD- non-Rheumatic heart disease

Age group	Cause		Total (%)
	RHD (%)	Non-RHD (%)	
≤30 Year	7 (5.78)	1 (1.96)	8 (4.65)
31-40 Year	13 (10.74)	3 (5.88)	16 (9.3)
41-50 Year	33 (27.27)	9 (17.64)	42 (24.4)
51-60 Year	41 (33.88)	11 (21.57)	52 (30.23)
61-70 Year	25 (20.66)	18 (35.29)	43 (25.0)
>70 Year	2 (1.65)	9 (17.65)	11 (6.39)
Total patients	121	51	172

Clinical profile in atrial fibrillation

Four patients were smokers, eight patients were alcoholic, five patients had diabetes mellitus, 20 patients had hypertension, 10 patients had cardiomyopathy, eight patients had prior coronary artery disease, nine patients had a history of chronic obstructive airway disease and one patient had a history of heart failure as shown in Table 2.

**Table 2:** Clinical profile in atrial fibrillation RHD- Rheumatic heart disease, Non-RHD- non-Rheumatic heart disease

Patients' history	Cause		Total N(%)
	RHD N(%)	Non-RHD N(%)	
Smoking	1 (0.8)	3 (5.9)	4 (2.32)
Alcohol	2 (1.6)	6 (11.8)	8 (4.65)
Diabetes	0 (0)	5 (9.8)	5 (2.9)
Hypertension	0 (0)	20 (39.21)	20 (11.6)
Cardiomyopathy	0 (0)	10 (19.6)	10 (5.8)
Heart failure	0 (0)	1 (1.96)	1 (0.8)

Left atrial function

The mean left atrial diameter of the study population was 50.5±9.5 mm. The Minimum left atrial diameter was 31 mm and the maximum left atrial diameter was 101 mm. The mean left atrial diameter was significantly higher in RHD patients than in Non-RHD patients (52.08± 10.13 vs 46.7±6.78 mm, p=0.001) as shown in Table 3. The mean left atrial maximal volume in our population was 168.6±58.5 mL. Mean maximum atrial volume was significantly higher in RHD patients as compared to Non-RHD patients (173.5±56.3 vs 153.96±39.8 mL, p=0.025). The mean left atrial minimal volume in our population was 111.1±39.9 mL. Mean minimum left atrial volume was higher in RHD patients as compared to Non-RHD patients (117.45±41.62 vs 98.04±23.51 mL, p=0.002) as shown in Table 3. The mean left atrial ejection fraction in the study population was 34.1±5.2%. The minimum left atrial ejection fraction was 21% and the maximum left atrial ejection fraction was 48%. Mean ejection fraction was significantly lower in the RHD group of patients as compared to the Non-RHD group of patients (33.53±5.06% vs 35.49±5.40%, p=0.024) as shown in Table 3.

**Table 3:** Left atrial and LAA function in the study population RHD- Rheumatic heart disease, Non-RHD- non-Rheumatic heart disease, LAA- Left atrial appendage

Parameters studied		Cause	Mean ± SD	p-Value
Left atrial diameter (mm)		RHD	52.08 ± 10.13	0.001
		Non-RHD	46.67± 6.78	
Mean left atrial volume (mL)	Maximum volume (mL)	RHD	173.5±56.3	0.025
		Non-RHD	153.96±39.8	
	Minimum volume (mL)	RHD	117.45±41.62	0.002
		Non-RHD	98.04±23.51	
Left atrial ejection fraction (%)		RHD	33.53±5.06	0.024
		Non-RHD	35.49± 5.40	
LAA orifice area (mm^2^)		RHD	7.52±1.22	0.005
		Non-RHD	6.94±1.17	
LAA emptying velocity (mL/s)		RHD	20.49±3.95	0.002
		Non-RHD	22.8±5.96	

Left atrial appendage function

The mean left atrial appendage (LAA) orifice area of the study population was 7.2±1.2 mm^2^. The minimum LAA orifice area was 4.8 mm^2^ and the maximum LAA orifice area was 12.0 mm^2^. The mean left atrial appendage orifice area in RHD patients was 7.52±1.22 mm^2^. The mean left atrial appendage orifice area in Non-RHD patients was 6.94±1.17 mm^2^. The mean orifice area of RHD patients was significantly higher than the Non-RHD patients (7.52±1.22 vs 6.94±1.17 mm^2^, p=0.005) as shown in Table 3. The mean left atrial appendage emptying velocity in the study population was 20.8±5.2 mL/s. The minimum LAA emptying velocity was 9.2 mL/s and the maximum LAA emptying velocity was 46.0 mL/s. Mean LAA emptying velocity was significantly lower in RHD patients as compared to Non-RHD patients (20.49±3.95 vs 22.8±5.96 mL/s, p=0.002) as shown in Table 3 above. 

## Discussion

Cardioembolic strokes secondary to atrial fibrillation accounts for 15% of all ischaemic strokes [5]. Left atrial structure and function increasingly become abnormal with a greater electrical burden of atrial fibrillation and associated with higher stroke risk as estimated by the CHADS2 score [6]. Moreover, left atrial dysfunction is present despite normal left atrium size and sinus rhythm, suggesting that the assessment of left atrium function may add important incremental information in the evaluation of atrial fibrillation patients [6]. 

Several studies have shown that increased left atrium dimensions, the presence of spontaneous echo contrast, and LAA thrombus and LAA flow peak velocity are associated with increased risk of stroke in atrial fibrillation patients [7-9]. In a study by Boldt et al., an assessment of fibrosis in left atrial tissue of patients with atrial fibrillation with and without underlying mitral valve disease revealed that there is an increase in protein concentration, especially collagen I, in patients with atrial fibrillation as compared to patients with sinus rhythm. Collagen III was not significantly increased in lone atrial fibrillation but was significantly increased in atrial fibrillation combined with mitral valve disease, both compared with sinus rhythm (p = 0.01) [10].

The mean age of our study population was 54.11±12.3 years. In a similar study in India by Bhardwaj R [11] and Narasimhan et al. [12], the mean age was reported as 51.24 ± 15.36 years and 59.9 ±14.4 years respectively. The mean age of our study population was similar to the other Indian studies but comparatively higher than the mean age in the international studies. In India, rheumatic heart disease is still more prevalent as compared to the western world where non-valvular atrial fibrillation is more prevalent. In our study population with atrial fibrillation, rheumatic heart disease was the most common associated cardiovascular condition in 121 (70.3%) patients. However, in contrast to our study, other Indian studies had hypertension as the most common underlying cardiovascular condition (50.8%), followed by valvular heart disease (40.7%) [12].

In our study, the mean left atrial diameter in RHD patients was higher than the mean diameter in Non-RHD patients (52.08± 10.13 vs 46.7±6.78 mm, p=0.001). In the study by Bhardwaj R also, the mean left atrium size was higher in RHD patients (53.01±11.54 mm) as compared to the study population (47.80±12.25 mm) [11]. Left atrial volume is an important predictor of outcome and adverse events in patients with lone AF. Osranek et al. have found left atrium volume ≥32 mL/m^2^ to be an independent predictor of significantly worse event-free survival with increased cerebral infarctions [13]. In our study, mean left atrial maximal volume and mean left atrial minimum volume were significantly higher in RHD patients than in Non-RHD patients. Decreased LAA contraction velocity has been associated with an increased prevalence of thrombus formation, increasing the risk of ischaemic stroke. In the SPAF III (Stroke Prevention in Atrial Fibrillation III) trans-esophageal substudy, 17% of patients with LAA contraction velocities ≤20 cm/s had thrombi as compared to 5% of the patients with higher velocities [14]. The deranged left atrium and LAA function in patients with rheumatic heart disease might lead to poorer outcomes and more adverse events in these patients.

Limitations

The study is a cross-sectional, observational study with a small sample size and does not include follow-up of patients to look for clinical outcomes in patients with deranged left atrial and LAA function. Moreover, strain or three-dimensional trans-esophageal echocardiography was not done in our study which would have given a better insight into atrial function.

## Conclusions

Rheumatic heart disease is still the most common cause of atrial fibrillation in developing nations. The left atrium and LAA functions are more deranged in patients with atrial fibrillation and rheumatic heart disease, leading to a possible poor cardiovascular outcome. More standardization and large follow-up studies are required to assess clinical outcomes in these patients.
